# Early liver function improvement following successful treatment of chronic hepatitis C in patients with decompensated cirrhosis: a real-life study

**DOI:** 10.6061/clinics/2021/e3186

**Published:** 2021-11-10

**Authors:** Mariana Sandoval Lourenço, Patricia Momoyo Y. Zitelli, Marlone Cunha-Silva, Arthur Ivan N. Oliveira, Roque Gabriel Rezende de Lima, Souza Evandro de Oliveira, Claudia P. Oliveira, Tiago Sevá-Pereira, Flair J. Carrilho, Mario G. Pessoa, Daniel F. Mazo

**Affiliations:** I Divisao de Gastroenterologia (Gastrocentro), Faculdade de Ciencias Medicas, Universidade Estadual de Campinas (UNICAMP), Campinas, SP, BR.; II Divisao de Gastroenterologia e Hepatologia Clinica, Departamento de Gastroenterologia, Hospital das Clinicas (HCFMUSP), Faculdade de Medicina, Universidade de Sao Paulo, Sao Paulo, SP, BR.

**Keywords:** Hepatitis C, Liver Cirrhosis, Antiviral Agents, Treatment Outcome, Safety

## Abstract

**OBJECTIVES:**

Despite higher rates of sustained virologic response (SVR), important concerns remain when patients with decompensated cirrhosis due to hepatitis C virus (HCV) are treated with direct-acting antiviral agents (DAA). Questions include efficacy, safety, and the magnitude of liver function improvement. Here, we aimed to evaluate HCV treatment data in this specific population in Brazil.

**METHODS:**

We included 85 patients with decompensated cirrhosis submitted to HCV therapy with DAA followed at two academic tertiary centers in the southeastern region of Brazil.

**RESULTS:**

Seventy-nine patients (92.9%) were Child-Pugh (CP) score B, and six (7.1%) were CP score C. The mean MELD score was 12.86. The most common treatment was sofosbuvir plus daclatasvir±ribavirin for 24 weeks. The overall intention-to-treat (ITT) SVR rate was 87.4% (74/85) and modified-ITT 96.1% (74/77). ITT SVR was associated with lower baseline INR values (*p*=0.029). Adverse events (AE) occurred in 57.9% (44/76) of patients. Serious AE were reported in 12.8% (10/78), and were related to the presence of hepatic encephalopathy (*p*=0.027). SVR was associated with improvement in CP (*p*<0.0001) and MELD scores (*p*=0.021). Among baseline CP score B patients with SVR, 46% (29/63) regressed to CP score A. Ascites was independently associated with no improvement in liver function in patients who achieved SVR (*p*=0.001; OR:39.285; 95% CI:4.301-258.832).

**CONCLUSIONS:**

Patients with decompensated HCV cirrhosis showed a high SVR rate with interferon-free therapy. Early liver function improvement occurred after successful HCV eradication. However, long-term follow-up of these patients after SVR remains strongly advised.

## INTRODUCTION

Chronic hepatitis C virus (HCV) infection remains one of the main causes of liver disease worldwide affecting 71 million people globally (1). In Brazil, it is estimated that 0.53% of the total population has antibodies against HCV (2). If untreated, 15-35% of these patients could progress to cirrhosis over a 25-to-30-year period, putting them at high risk of hepatic decompensation and death (3).

End-stage liver disease and hepatocellular carcinoma associated with chronic HCV infection are among the most common indications for liver transplantation in the United States, Europe, and Brazil (4-7). Fortunately, this scenario is changing drastically with the widespread use of direct-acting antiviral agents (DAA) that lead to successful HCV eradication (4-6).

HCV sustained virologic response (SVR) is associated with mortality reduction due to liver and non-liver related causes (8). Its benefits have also been described in patients with decompensated cirrhosis (5,9). However, important concerns remain when treating patients with HCV-related end-stage liver disease. The optimal time for treatment, the efficacy, and the safety of each regimen are relevant topics to consider in this special population (10). Furthermore, data regarding hepatitis C treatment in large cohorts of decompensated patients are rare in Brazil. In a national study conducted by the Brazilian Society of Hepatology, the authors reported lower SVR rates in subjects with decompensated cirrhosis than those with compensated cirrhosis (11). The magnitude of the benefit of successful HCV eradication in advanced decompensated cirrhosis, especially in Child-Pugh (CP) score C patients, is also an area of uncertainty (12). In addition, data on the efficacy and safety of HCV treatment in this population could help define and implement more assertive public health care policies.

In this context, this study describes the efficacy, safety, and factors associated with liver function improvement in patients with decompensated HCV cirrhosis treated with DAA in two public tertiary academic centers in southeastern Brazil.

## METHODS

### Clinical Design and Patients’ Selection

This is a multicenter retrospective study conducted between November 2015 and November 2019 at the Outpatient Units of the Division of Gastroenterology (Gastrocentro) of the University of Campinas (UNICAMP) and the Department of Gastroenterology at Hospital das Clinicas of University of Sao Paulo School of Medicine (FMUSP). The target population was patients with decompensated cirrhosis due to chronic hepatitis C who received treatment with DAA.

The inclusion criteria were age ≥18 years old, the presence of chronic hepatitis C as defined by HCV polymerase chain reaction (PCR) RNA positivity for at least 6 months, decompensated liver disease defined by a CP score B or C, and treatment with interferon-free HCV regimen. Exclusion criteria were other diagnosed chronic liver diseases, HIV infection, hepatitis B infection, active hepatocellular carcinoma (HCC), previous liver transplantation, previous interferon-free HCV treatment, and lack of information regarding the current HCV treatment regimen. Previous alcohol intake (>100 g/week) was allowed.

### HCV treatment regimen

All patients received at least one dose of the prescribed HCV treatment that was obtained in accordance with the 2015 Brazilian Ministry of Health’s Therapeutic Guidelines for decompensated cirrhosis (13). Briefly, for genotype 1 HCV patients, the recommended regimen was a combination of sofosbuvir (SOF) plus daclatasvir (DCV) ± ribavirin (RBV) for 24 weeks. In genotype 3, the same combination was recommended for 12 weeks. Subsequently, a ministerial decree extended HCV genotype 3 treatment to 24 weeks (14). Although SOF plus simeprevir (SMV) was approved for genotype 1 HCV patients with compensated disease (13), four patients received this regimen according to the assistant physicians’ discretion and were included in this study.

### Variables evaluated

Demographic and anthropometric data (age, gender, and body mass index) were obtained from the medical charts as well as the presence of comorbidities [arterial hypertension, diabetes mellitus, dyslipidemia, hypothyroidism, psychiatric disorder, and previous alcoholism]. Liver complications (ascites, esophageal varices, portal hypertensive bleeding, hepatic encephalopathy, and previous HCC) were registered. Data regarding previous interferon-based HCV treatment were also registered (pegylated interferon±boceprevir or telaprevir).

Serum biochemistry tests included alanine aminotransferase (ALT), aspartate aminotransferase (AST), total bilirubin, creatinine, albumin, platelet count, international normalized ratio (INR), hemoglobin, and alpha-fetoprotein. These were collected before HCV treatment initiation, and 12-24 weeks after treatment completion. CP and MELD scores were also evaluated. Serum HCV-RNA real-time PCR was performed with Amplicor HCV Monitor 2.0 (Abbott Molecular, Des Plaines, IL, USA, detection limit: 12 IU/mL). HCV genotyping was performed using Versant^®^ HCV Genotype 2.0 (LiPA) (Imunogenetics, Ghent, Belgium).

### HCV treatment outcome and efficacy analysis

SVR was defined by an HCV RNA level below the limit of detection 12-24 weeks after treatment completion. For treatment efficacy evaluation, an intention-to-treat (ITT) analysis was performed, and counted all patients treated and included in the study. Patients lost to follow-up or without SVR information were considered non-responders. In the second approach, patients lost to follow-up, who discontinued treatment, or died (treatment unrelated death) were excluded from the efficacy analysis. This approach was defined as modified intention-to-treat (m-ITT) analysis.

### HCV treatment safety analysis

Adverse events (AE) were classified in accordance with the adapted regulation of the Common Terminology Criteria for Adverse Events (CTCAE) (15). Anemia was classified as grade 1 (Hb 10-8g/dL), grade 2 (Hb<8 or need for blood transfusion), grade 3 (risk of death), and grade 4 (death). Serious adverse events (SAE) were considered as: 1- worsening of the liver decompensation; 2- need for hospitalization; 3- need to discontinue treatment; and 4- events that resulted in death.

### Ethical considerations

The Ethics Committee of the University of Campinas and of the Hospital das Clinicas of the University of Sao Paulo School of Medicine approved this study (numbers 2,042,967 and 2,670,862, respectively).The protocol was conducted in accordance to the ethical guidelines of the 2013 World Medical Association Declaration of Helsinki. Informed consent was waived.

### Statistical analysis

Frequency tables of the categorical variables with absolute frequencies (n) and percentages (%) as well as descriptive statistics of the numerical variables, with means, and standard deviations (SD) were reported. The chi-square test and Fisher’s exact test were used to compare categorical variables; the Mann-Whitney test was used for numerical variables. The maximum percentage of missing values for any baseline and post treatment clinical/biochemical covariate were 15.2% and 22.3%, respectively. AE and SAE covariates with missing values were 9 and 7, respectively. The Wilcoxon test and McNemar test were used to analyze liver function improvement in patients who achieved SVR. Logistic regression analysis was performed whenever possible. A stepwise variable selection approach was used in the multivariate logistic regression analysis. The odds ratio (OR) and 95% confidence interval (CI) were calculated. A probability value of <0.05 was considered significant. Statistical Analysis System (SAS) for Windows software package, version 9.4 (SAS Institute Inc, 2002-2008, Cary, NC, USA) was used for the statistical analyses by the Statistics Service at School of Medical Sciences of the University of Campinas.

## RESULTS

### Baseline characteristics

A total of 85 patients with chronic hepatitis C and decompensated cirrhosis who were submitted to DAA treatment during the study period were included. The main baseline demographic, clinical, and laboratory characteristics of the study population are shown in Table 1. The mean age was 56.13 years. The most common comorbidities were arterial hypertension and diabetes mellitus; 15.7% of the individuals had alcohol as a cofactor for their liver disease. The most common HCV genotype was genotype 1 in 75.4% of the subjects. Regarding baseline liver function, seventy-nine (92.9%) of the patients had CP score B, and six (7.1%) had CP score C. The mean CP and MELD scores were 7.89 and 12.86, respectively. The median MELD score was 13 (minimum 6, maximum 23). The most frequent liver decompensation event in the cohort was ascites and was detected in 71.8% of the cases.

### Therapeutic regimens and efficacy analysis

SOF+DCV+RBV was used in 60 (70.6%) patients, SOF+DCV in 21 (24.7%) patients, SOF+SMV+RBV in 3 (3.5%) cases, and one patient (1.2%) received SOF+SMV. Twenty-four (28.2%) treatments had a 12-week duration while 61 (71.8%) were for 24 weeks. RBV was used in 63 (74.1%) of the treatments. The mean daily dose of ribavirin was 11.57±3.01 mg/kg. The overall SVR rate was 87.4% (74/85) in the ITT analysis, and 96.1% (74/77) by m-ITT. Eight (9.4%) patients had no HCV-RNA information or were lost to follow-up 12-24 weeks after treatment precluding SVR analysis. The ITT and m-ITT SVR in genotype 1 patients were 89% (57/64) and 96.6% (57/59), respectively. Genotype 3 patients were 80.9% (17/21) and 94.4% (17/18). All patients treated with the SOF+SMV±RBV achieved SVR. Likewise, all patients with CP C (6 patients) attained SVR. Table 2 shows the SVR rates according to the different therapeutic regimens, genotypes, and CP score.

There were no differences between ITT SVR rates regarding HCV genotype (*p*=0.453), CP score (*p*=1.0), previous HCV treatment (*p*=0.503), presence of ascites (*p*=0.496), hepatic encephalopathy (*p*=0.198), previous portal hypertension related bleeding (*p*=1.0), or previous HCC (*p*=1.0). The only baseline variable associated with ITT SVR was lower INR values (*p*=0.029), as shown in Table 3. Logistic regression analysis was not possible due to the lower number of non-SVR patients in the cohort.

### Safety analysis

AE were described in 57.9% (44/76) of the study population. The most common were fatigue (43.4%), anemia (34.2%, mainly grade 1 and 2), headache (18.4%), nausea (13.1%), itching (9.2%), insomnia (7.8%), and skin rash (7.8%). Infection occurred in 12.7% (8/63) of the patients. SAE were reported in 12.8% (10/78) of the cases. Six patients had worsening of liver decompensation, and three patients died during treatment (3.8%) (Table 4). The occurrence of AE during treatment was associated with a higher CP score at baseline (*p*=0.045), higher daily dose of RBV (12.43±3.27 *vs* 10.20±2.24 mg/kg, *p*=0.010), and prior hepatic encephalopathy (*p*=0.015). In the univariate logistic regression analysis, the variables associated with AE were the presence of hepatic encephalopathy (*p*=0.018; OR:3.611; 95% CI:1.241-10.504), baseline CP score (*p*=0.045; OR:1.691; 95% CI:1.011-2.830), and RBV daily dose (*p*=0.024; OR:1.306; 95% CI:1.035-1.648). Baseline serum albumin (*p*=0.006; OR:0.093; 95% CI:0.017-0.509) and AST values (*p*=0.007; OR:0.976; 95% CI:0.958-0.993) were independently associated with the occurrence of AE (Table 5). AE was more likely to occur during treatment if these values were lower. The only variable associated with the occurrence of SAE in the cohort was the presence of hepatic encephalopathy prior to treatment (*p*=0.027). Logistic regression analysis regarding SAE was not statistically feasible.

### Evaluation of serum exams and liver function improvement after successful HCV treatment

**Figure 1:**
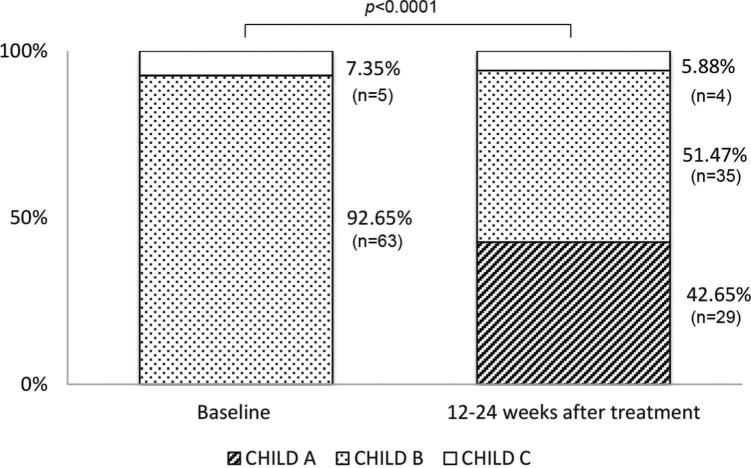
Evolution of Child-Pugh score after successful therapy.

In patients with SVR, we evaluated the evolution of the CP score 12-24 weeks after completion of treatment and 68 patients of the 74 that had this information available. Among the 63 patients with a baseline CP score B, 46% (29) returned to CP score A and 54% (34) remained CP score B after treatment. Among the CP score C patients who achieved SVR (5 individuals), only one patient (20%) had improved liver function returning to CP score B. (Figure 1). In the whole cohort, the improvement in CP score was significant (*p*<0.0001). We also observed a significant reduction in MELD score (*p*=0.021; median decrease: 1.0). There was a reduction in bilirubin (*p*<0.0001), alpha-fetoprotein (*p*<0.0001), ALT (*p*<0.0001), and AST levels (*p*<0.0001). Moreover, an elevation of serum albumin levels (*p*=0.0001) was noticed after SVR. In the univariate logistic regression analysis, the baseline factors associated with no CP score improvement were the presence of ascites (*p*=0.001; OR:8.167; 95% CI:2.305-28.940), lower serum albumin (*p*=0.031; OR: 0.288; 95% CI:0.093-0.894), and higher INR (*p*=0.022; OR:44.264; 95% CI:1.708- non calculable). In the multivariate logistic regression analysis, the presence of ascites was associated with a 39.28-fold increased finding of no liver function improvement in those with SVR (*p*=0.001; OR:39.285; 95% CI: 4.301-258.832) as shown in Table 6.

## DISCUSSION

Our study population consisted of decompensated cirrhotic patients: These were mainly individuals with CP score B with a mean MELD score of 12.86. This patient profile is similar to other real-life studies that evaluated DAA in decompensated cirrhosis (16,17). Our main HCV treatment regimen was SOF plus DCV plus RBV. ALLY-1 is an open-label phase 3 trial that evaluated 60 patients with advanced cirrhosis; 80% of them had decompensated disease using the same DAA combination of our study for 12 weeks (18). The reported SVR was 83%, which is slightly lower than the 87.4% achieved here. This difference could be, at least in part, attributed to the longer duration of our HCV treatment and to the higher daily dose of RBV of our patients. Longer treatment duration and adjuvant RBV are known to increase the likelihood of SVR, especially in decompensated cirrhosis and genotype 3 patients (19). Four patients were treated with the combination of SOF plus SMV, and all of them achieved SVR. Although SMV is not recommended in patients with CP score ≥B (13), the real-world safety and efficacy data of SOF+SMV have already been described in this context (20,21).

HCV-decompensated liver disease negatively impacts SVR rates in comparison to compensated and non-cirrhotic states (22). The HCV-LALREAN cohort in Latin America also described higher DAA treatment failure rates in patients with decompensated cirrhosis in multivariate logistic regression analysis (23). The Brazilian Society of Hepatology study gathered 19 HCV treatment centers with 3939 patients: The authors showed lower SVR rates in the 108 individuals with decompensated cirrhosis versus those with compensated cirrhosis (75% *vs* 91.5%, *p*<0.001, respectively) (11).

Within clinical trials, the SVR rates in decompensated cirrhosis with different DAA regimens range from 72% to 94%, but the majority of patients had CP score B with limited data for CP score C patients (18,24,25). In real-life studies, the reported SVR rates range from 75% to 96.7% (11,12,16,17,26-29). These SVR rates are quite similar to the ones reported in clinical trials and in the present study. HCV therapy in sicker patients can be challenging. Sandmann et al. (30) reported the HCV therapy experience in 30 patients with a MELD score ≥15 at inclusion (mean 18.5) from five German transplant centers. The overall SVR rate was 73.3%, and five patients (16.7%) died due to liver-related causes (30). In our study population, the only baseline variable associated with SVR was lower INR values, i.e., patients with better liver function. Less severe liver disease, represented by lower CP scores and serum bilirubin levels, or higher serum levels of albumin are consistently associated with a higher chance of achieving SVR in decompensated patients (28,29). Despite several published reports showing lower SVR rates in patients with HCV genotype 3 (11,26,28), this was not found in our study, possibly due to the limited number of patients enrolled (n=21).

SAE were reported in 12.8% of our study population, and its occurrence was associated with prior hepatic encephalopathy. Tahata et al. (17) described 3.6% deaths among 82 HCV decompensated cirrhotic subjects similar to our mortality rate (3.8%). Miotto et al. (31) described 32.2% of premature treatment interruption among 31 Brazilian patients with cirrhosis Child Pugh B or C, associated with higher MELD scores.

Early liver function improvement with successful HCV eradication was a remarkable finding in this study. Almost 50% of CP score B patients regressed to a compensated state of cirrhosis in short-term follow-up. Compelling data have already been described regarding liver function improvement following SVR. An integrated analysis of data from four clinical trials of sofosbuvir-based interferon-free therapy in 528 patients with decompensated cirrhosis who achieved SVR showed that 31.6% of patients with baseline CP score B and 12.3% of patients with CP score C regressed to CP score A at week 36 after the initiation of HCV treatment (27). In our cohort, no CP score C patient became CP score A, and one patient (20%) returned to a CP score B. Debnath et al. (12) evaluated 62 patients with decompensated cirrhosis due to HCV in India and reported that 54.8% attained a CP score A after treatment. Similar to our findings, none of the patients with a CP score C became a CP score A. The switch to compensated cirrhosis from CP score B has been described in 31.6% to 61.8% of the patients after successful DAA therapy in recent real-life studies (12,16,17,27-29). However, the percentage of patients that turned back to a compensated state from CP score C are much lower, ranging from 0% to 12.3% (12,17,27). In our cohort, baseline presence of ascites, lower serum albumin, and higher serum INR levels were associated with the absence of liver function improvement in the univariate logistic regression analysis. Ascites remained significant in the multivariate analysis. Foster et al. (26) evaluated 409 HCV decompensated cirrhotic patients in the United Kingdom and also found an association between higher serum albumin levels and greater likelihood to benefit from therapy. Gheorghe et al. (29) in Romania reported that serum albumin >3.5 g/dL had an OR of 3.317 for liver function improvement. In addition, pooled data analyses from clinical trials with SOF-based therapy in decompensated cirrhosis corroborate the association of higher baseline serum albumin and liver function improvement (27). Serum albumin >3.5 g/dL is a component of the BE3A score for prediction of treatment benefit (27). The absence of ascites before DAA-induced SVR is another component of the BE3A score (27). In line with these results, we also found that the presence of ascites at baseline was independently associated with the lack of liver function improvement in patients with SVR.

The magnitude of liver function improvement should also be noted. The median MELD score reduction in our patients was just 1.0, which concurs with Foster et al. (26) in the United Kingdom. Interestingly, the HCV-TARGET cohort study showed that the overall mean changes in MELD, total bilirubin, and albumin were marginal after a median follow-up of 4 years after treatment (32). In addition, Bittermann et al. (33) recently reported a retrospective study using national US data and found that the delisting of HCV patients because of clinical improvement increased, but still remains infrequent in the DAA era. These patients may remain at high risk of decompensation—some of them in the “MELD purgatory;” therefore, they must continue to be monitored after HCV therapy (32,34,35).

This study does have some limitations. This is a retrospective study, and thus data was obtained from medical records with missing information especially regarding AE. In addition, it was not possible to reliably assess the patient's adherence to treatment. Finally, we had a moderate number of included patients and a short follow-up evaluation. Even so, this study encompassed one of the largest cohorts of Brazilian subjects with decompensated cirrhosis treated with DAA.

In conclusion, we showed that a SVR rate above 87% could be obtained in a real-life study of HCV patients with decompensated cirrhosis. This was associated with lower baseline INR values. SAE occurred in 12.8% of the patients and were related to prior hepatic encephalopathy. In a short-term follow-up, almost half of the CP score B patients with SVR regressed to a compensated state of cirrhosis, and the absence of liver function improvement was associated with the presence of ascites prior to treatment. Long-term follow-up of patients with HCV end-stage liver disease after SVR is warranted. These findings may be useful for physicians to predict the response of decompensated cirrhotic patients to DAA therapy and thus can facilitate the decision-making process.

## AUTHOR CONTRIBUTIONS

Mazo DF conceived and designed the study, took care of the patients, contributed to the data analysis and interpretation and wrote the manuscript. Lourenço MS collected and assembled the data, took care of the patients, and contributed to the data analysis and interpretation. Zitelli PMY and Oliveira AIN collected and assembled the data, and took care of the patients. Lima RGR, Souza EO and Sevá-Pereira T took care of the patients. Cunha-Silva M, Oliveira CP, Carrilho FJ and Pessoa MG took care of the patients, and critically reviewed the manuscript. All authors critically revised the manuscript, approved the final version to be published, and agree to be accountable for all aspects of the work.

## Figures and Tables

**Table 1:** Demographic, clinical and biochemical characteristics of patients with HCV (n=85).

	HCV patients (n=85) % (n) or mean±SD
Age (years)	56.13±11.14
Men / Women	50.6% (43) / 49.4% (42)
Type 2 diabetes	34.9% (29/83)
Dyslipidemia	21.7% (18/83)
High-blood pressure	41.0% (34/83)
BMI (kg/m^2^) (n=72)	27.27±5.16
Hypothyroidism	13.3% (11/83)
Psychiatric disorder	10.7% (9/84)
Previous alcohol use	15.7% (13/83)
HCV genotype (n=85)	
1A	50.6%
1B	22.4%
1A/ 1B	2.4%
3	24.7%
HCV viral load (log IU/mL)	5.37±0.83
Previous HCV treatment (n=83)	
None	45.8% (38)
Peg-IFN + RBV	49.4 (41)
Peg-IFN + PI	4.8% (4)
Liver-related complications (n=85)	
Ascites	71.8% (61)
Esophageal varices	88.2% (75)
Portal hypertensive bleeding	27.4% (23)
Hepatic encephalopathy	36.5% (31)
Previous HCC (n=71)	11.3% (8)
AST (U/L)	73.27±39.27
ALT (U/L)	49.46±29.21
Total bilirubin (mg/dL)	2.15±1.54
Albumin (g/dL)	3.09±0.48
Platelets (/mm^3^)	77.01±41.93
INR	1.37±0.22
Creatinine (mg/dL)	0.99±0.95
Hemoglobin (g/dL)	12.14±2.15
Alpha-fetoprotein (ng/mL)	16.77±29.42
Child-Pugh classification (%)	
B	92.9% (79)
C	7.1% (6)
Child-Pugh Score	7.89±1.02
MELD Score	12.86±3.41

ALT: alanine aminotransferase; AST: aspartate aminotransferase; BMI: body mass index; HCC: hepatocellular carcinoma; HCV: hepatitis C virus; INR: international normalized ratio; MELD: Model for End-Stage Liver Disease; Peg-IFN: pegylated-interferon; PI: protease inhibitor (boceprevir or telaprevir); RBV: ribavirin; SD: standard deviation.

**Table 2:** SVR rates according to therapeutic regimens, HCV genotypes and CP Score.

SVR	ITT % (n)	m-ITT % (n)
Global	87.4% (74/85)	96.1% (74/77)
Genotype		
1	89% (57/64)	96.6% (57/59)
3	80.9% (17/21)	94.4% (17/18)
Treatment regimen		
SOF + DCV	71.4% (15/21)	93.7% (15/16)
SOF + DCV + RBV	91.6% (55/60)	96.4% (55/57)
SOF + SMV	100% (1/1)	100% (1/1)
SOF + SMV + RBV	100% (3/3)	100% (3/3)
Child-Pugh Score		
B	86% (68/79)	95.7% (68/71)
C	100% (6/6)	100% (6/6)

CP: Child-Pugh Score; DCV: daclatasvir; HCV: hepatitis C virus; ITT: intention-to-treat; m-ITT: modified intention-to-treat; RBV: ribavirin; SMV: simeprevir; SOF: sofosbuvir; SVR: sustained virologic response.

**Table 3:** Variables associated with SVR by ITT analysis (n=85).

	SVR (n=74) % (n) or mean±SD	Non-SVR (n=11) % (n) or mean±SD	*p* value
Age (years)	55.82±10.59	58.18±14.73	0.550
Men / Women	51.4% (38) / 48.6% (36)	45.5% (5) / 54.5% (6)	0.715
Type 2 diabetes	35.6% (26/73)	30.0% (3/10)	1.000
Dyslipidemia	19.2% (14/73)	40.0% (4/10)	0.212
High-blood pressure	37.0% (27/73)	70.0% (7/10)	0.082
BMI (kg/m^2^) (n=72)	27.44 ± 5.32	25.66 ± 3.18	0.470
Hypothyroidism	12.3% (9/73)	20.0% (2/10)	0.615
Psychiatric disorder	11.0% (8/73)	9.1% (1/11)	1.000
Previous alcohol use	16.4% (12/73)	10.0% (1/10)	1.000
HCV genotype (n=85)			
1	77.0%	63.6%	0.453
3	23.0%	36.4%	
Previous HCV treatment			
None	45.2% (33/73)	50.0% (5/10)	0.503
Peg-IFN + RBV	50.7 (37/73)	40.0 (4/10)	
Peg-IFN + PI	4.1% (3/73)	1.0% (1/10)	
Liver-related complications			
Ascites	73.0% (54/74)	63.6% (7/11)	0.496
Esophageal varices	89.2% (66/74)	81.8% (9/11)	0.611
Portal hypertensive bleeding	27.4% (20/73)	27.3% (3/11)	1.000
Hepatic encephalopathy	33.8% (25/74)	54.5% (6/11)	0.198
Previous HCC	11.3% (7/62)	11.1% (1/9)	1.000
AST (U/L)	72.28±39.59	80.40±38.09	0.431
ALT (U/L)	48.85±28.37	53.90±36.17	0.665
Total bilirubin (mg/dL)	2.17±1.58	1.99±1.24	0.815
Albumin (g/dL)	3.10±0.50	3.03±0.26	0.874
Platelets (/mm^3^)	76.91±43.37	77.70±31.44	0.566
INR	1.34±0.17	1.58±0.37	0.029*
Creatinine (mg/dL)	1.02±1.01	0.80±0.20	0.624
Hemoglobin (g/dL)	12.23±2.17	11.49±2.01	0.349
Alpha-fetoprotein (ng/mL)	16.92±30.22	15.66±24.39	0.781
Child-Pugh classification (%)			
B	91.9% (68/74)	100% (11/11)	1.000
C	8.1% (6/74)	0.0% (0/11)	
Child-Pugh Score	7.88±1.03	8.00±1.00	0.643
MELD Score	12.69±3.46	14.22±2.82	0.085
Dose of ribavirin (mg/kg/day)	11.65±3.01	10.75±3.42	0.654

Chi-square test, Fisher's exact test, and Mann-Whitney test. ALT: alanine aminotransferase; AST: aspartate aminotransferase; BMI: body mass index; HCC: hepatocellular carcinoma; HCV: hepatitis C virus; INR: international normalized ratio; ITT: intention-to-treat; Peg-IFN: pegylated-interferon; PI: protease inhibitor (boceprevir or telaprevir); RBV: ribavirin; SD: standard deviation; SVR: sustained virologic response.

* *p-*value <0.05.

**Table 4:** Safety analysis.*

	% (n)
**Adverse events**	
Fatigue	43.4% (33)
Anemia	34.2% (26)
Headache	18.4% (14)
Nausea	13.1% (10)
Itching	9.2% (7)
Insomnia	7.8% (6)
Skin rash	7.8% (6)
**Serious adverse events**	
Worsening of liver decompensation	7.7% (6)
Death	3.8% (3)

* Adverse events and serious adverse events data were available for 76 and 78 patients, respectively. Some patients had more than one adverse event during treatment.

**Table 5:** Factors associated with the occurrence of adverse events during treatment (n=76).

	Univariate analysis			Multivariate analysis		
Variable	OR	95% CI	*p* value	OR	95% CI	*p* value
Age (years)	1.030	0.986-1.076	0.189			
Women / Men	1.923	0.763-4.844	0.165			
Type 2 diabetes	0.893	0.345-2.312	0.815			
Dyslipidemia	1.490	0.485-4.571	0.486			
High-blood pressure	1.591	0.626-4.043	0.329			
BMI	1.056	0.956-1.168	0.283			
Hypothyroidism	0.711	0.187-2.698	0.615			
Psychiatric disorder	0.698	0.143-3.411	0.657			
Previous alcohol use	2.210	0.537-9.098	0.272			
Previous HCV treatment	1.143	0.443-2.949	0.782			
Liver-related complications						
Ascites	0.933	0.341-2.554	0.893			
Esophageal varices	3.154	0.725-13.723	0.125			
Portal hypertensive bleeding	0.958	0.347-2.649	0.934			
Hepatic encephalopathy	3.611	1.241-10.504	0.018*			
Previous HCC	0.698	0.143-3.411	0.657			
AST	0.992	0.980-1.004	0.174	0.976	0.958-0.993	0.007*
ALT	0.989	0.973-1.005	0.183			
Total bilirubin	0.966	0.724-1.290	0.815			
Albumin	0.378	0.128-1.118	0.078	0.093	0.017-0.509	0.006*
Platelets	1.000	1.000-1.000	0.174			
INR	2.532	0.268-23.922	0.417			
Creatinine	1.011	0.632-1.616	0.964			
Hemoglobin	0.989	0.802-1.219	0.914			
Alpha-fetoprotein	0.984	0.963-1.005	0.125			
Child-Pugh Score	1.691	1.011-2.830	0.045*			
MELD Score	1.040	0.910-1.188	0.564			
Ribavirin dose	1.306	1.035-1.648	0.024*			

Logistic regression. ALT: alanine aminotransferase; AST: aspartate aminotransferase; BMI: body mass index; CI: confidence interval; HCC: hepatocellular carcinoma; HCV: hepatitis C virus; INR: international normalized ratio; MELD: Model for End-Stage Liver Disease; OR: odds ratio; SVR: sustained virologic response.

* *p* value <0.05.

**Table 6:** Factors associated with absence of Child-Pugh score improvement with SVR.

	Univariate analysis			Multivariate analysis		
Variable	OR	95% CI	*p* value	OR	95% CI	*p* value
Age (years)	1.020	0.972-1.070	0.428			
Women / Men	1.296	0.494-3.398	0.598			
Type 2 diabetes	1.531	0.537-4.365	0.425			
Dyslipidemia	1.939	0.532-7.070	0.315			
High-blood pressure	0.737	0.272-1.998	0.548			
BMI	0.994	0.905-1.092	0.899			
Hypothyroidism	0.947	0.230-3.893	0.939			
Psychiatric disorder	0.743	0.139-3.981	0.728			
Previous alcohol use	0.719	0.206-2.514	0.605			
Liver related complications						
Ascites	8.167	2.305-28.940	0.001*	39.285	4.301-358.832	0.001*
Esophageal varices	3.853	0.691-21.482	0.123			
Portal hypertensive bleeding	0.690	0.233-2.042	0.502			
Hepatic encephalopathy	2.667	0.886-8.027	0.081			
Previous HCC	0.525	0.106-2.594	0.429			
AST	1.003	0.991-1.016	0.599			
ALT	0.994	0.977-1.011	0.467			
Total bilirubin	1.338	0.914-1.958	0.133			
Albumin	0.288	0.093 -0.894	0.031*			
Platelets	1.000	1.000-1.000	0.645			
INR	44.264	1.708- ------	0.022*			
Creatinine	0.594	0.255-1.383	0.227			
Hemoglobin	0.988	0.791-1.234	0.915			
Alpha-fetoprotein	0.985	0.965-1.005	0.146			
Ribavirin dose	1.006	0.809-1.251	0.956			

Logistic regression. **Abbreviations:** ALT: alanine aminotransferase; AST: aspartate aminotransferase; BMI: body mass index; CI: confidence interval; HCC: hepatocellular carcinoma; INR: international normalized ratio; OR: odds ratio; SVR: sustained virologic response.

* *p* value <0.05.
